# Traumatic pseudoaneurysm of the brachiocephalic artery obstructing the airways

**DOI:** 10.1016/j.ijscr.2020.11.064

**Published:** 2020-11-24

**Authors:** Bader M. Tahlawi, Amirah Hassan, Mohamed Regal

**Affiliations:** a King Fahad Military Medical Complex, Dhahran, Eastern Province, Saudi Arabia; b KFHU (King Fahad Hospital of the University), Al Khobar, Eastern Province, Saudi Arabia; c Ministry of Health, Saudi Arabia; d Bahrain Defence Force Royal Medical Services, Military Hospital

**Keywords:** Mediastinal tumor, Pseudoaneurysm, Airway obstruction, Innominate artery, Brachiocephalic artery

## Abstract

This is a case report of a young patient referred to our ER as a case of mediastinal tumor compressing the airways. On presentation, the patient had severe stridor and tachypnea, with oxygen saturation less than 60%. As the patient presented alone to the ER, obtaining any history was not possible. Chest x-ray showed a huge right paratracheal mediastinal mass compressing the airway. The patient was not able to lie supine and was taken to the OR for intubation under fiberoptic bronchoscopy guidance. Bronchoscopic evaluation showed significant compression of the trachea from the right side. A small endotracheal tube (size 5) was successfully inserted. Then, the patient was taken for a chest CT with IV contrast. Chest CT showed a huge vascular mass compressing the trachea, the right mainstem bronchus, and the superior vena cava. The study showed the proximal innominate artery, followed by a pseudoaneurysm. There was an interruption of the right subclavian artery at its origin. Collaterals were seen supplying the right upper limb. Surgical resection was done. Postoperatively the patient gave a history of chest-penetrating injury in the right infraclavicular area, 15 years prior to presentation. The pseudoaneurysm of the innominate artery is a rare delayed complication of chest trauma.

## Highlights

• Pseudoaneurysm of the brachiocephalic artery is a very rare condition, with the majority occurring post chest trauma.
• The delayed presentations are usually nonspecific and may vary from mild to very severe and potentially life threating symptoms.
• CT angiogram is the most accurate diagnostic tool that sets the pathway for a well-planned surgical intervention.

## 1 Introduction

Aneurysms and pseudoaneurysms of the Innominate (Brachiocephalic) artery are extremely rare conditions. Traumatic injuries are the frequent cause [1]. The acute presentation of a thoracic great vessel injury is usually dramatic, with massive hemothorax and hemorrhagic shock [2]. The delayed presentations are usually nonspecific and may vary from mild respiratory distress mimicking bronchial asthma to severe airway obstruction [7], [8]. Other delayed manifestations may include dysphagia, hemoptysis, hematemesis, pulse and neurological deficits, bruits, or cardiac failure [3].

## 2 Case report

**Fig. 1:**
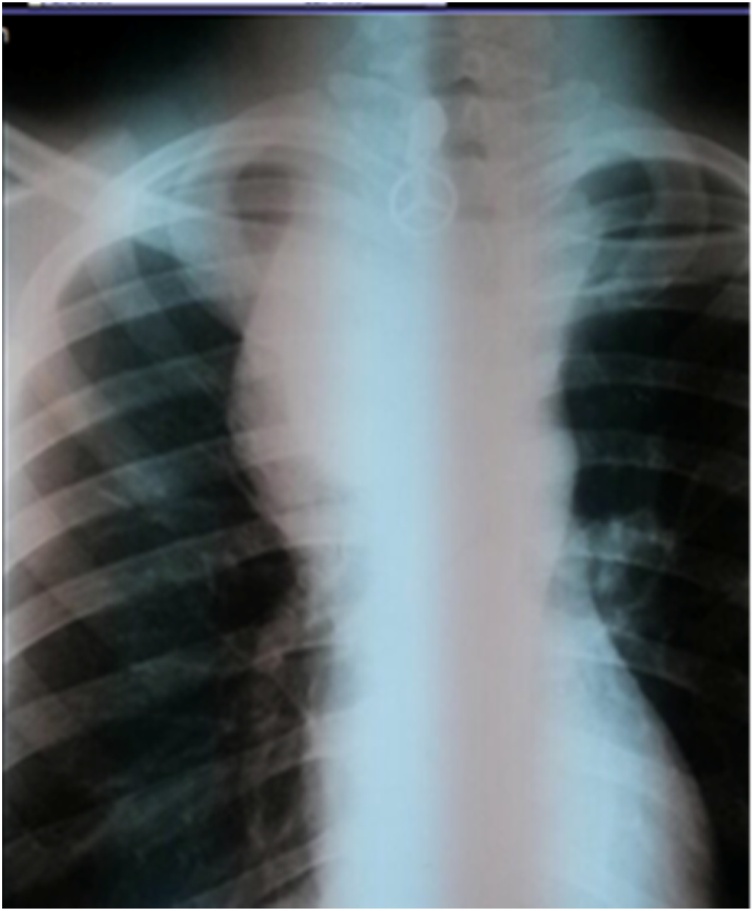
CXR on presentation, showing a huge right paratracheal mass obstructing the airways.

**Figs. 2 & 3:**
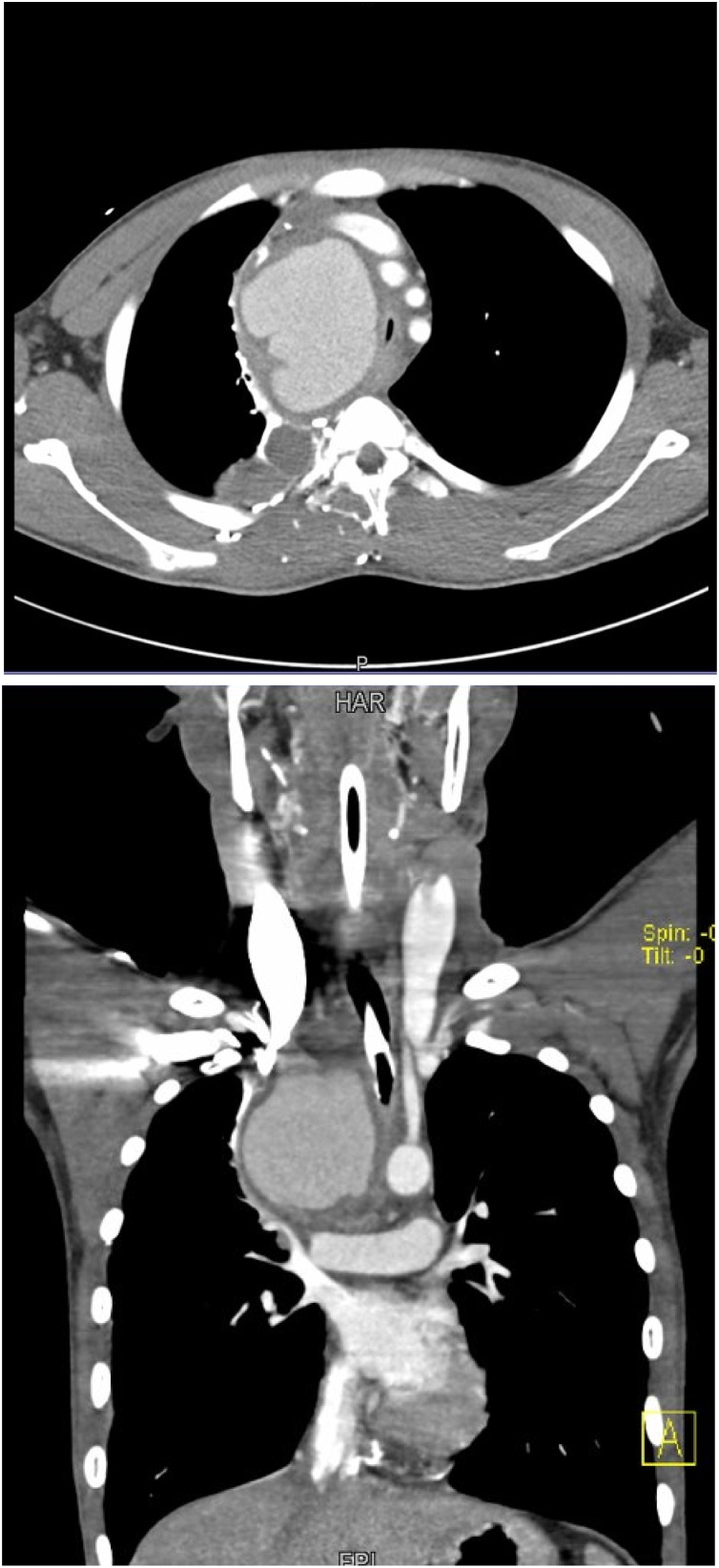
CT chest with IV contrast showing a huge mediastinal vascular mass compressing the airways significantly with contralateral mediastinal shift.

**Figs. 4 & 5:**
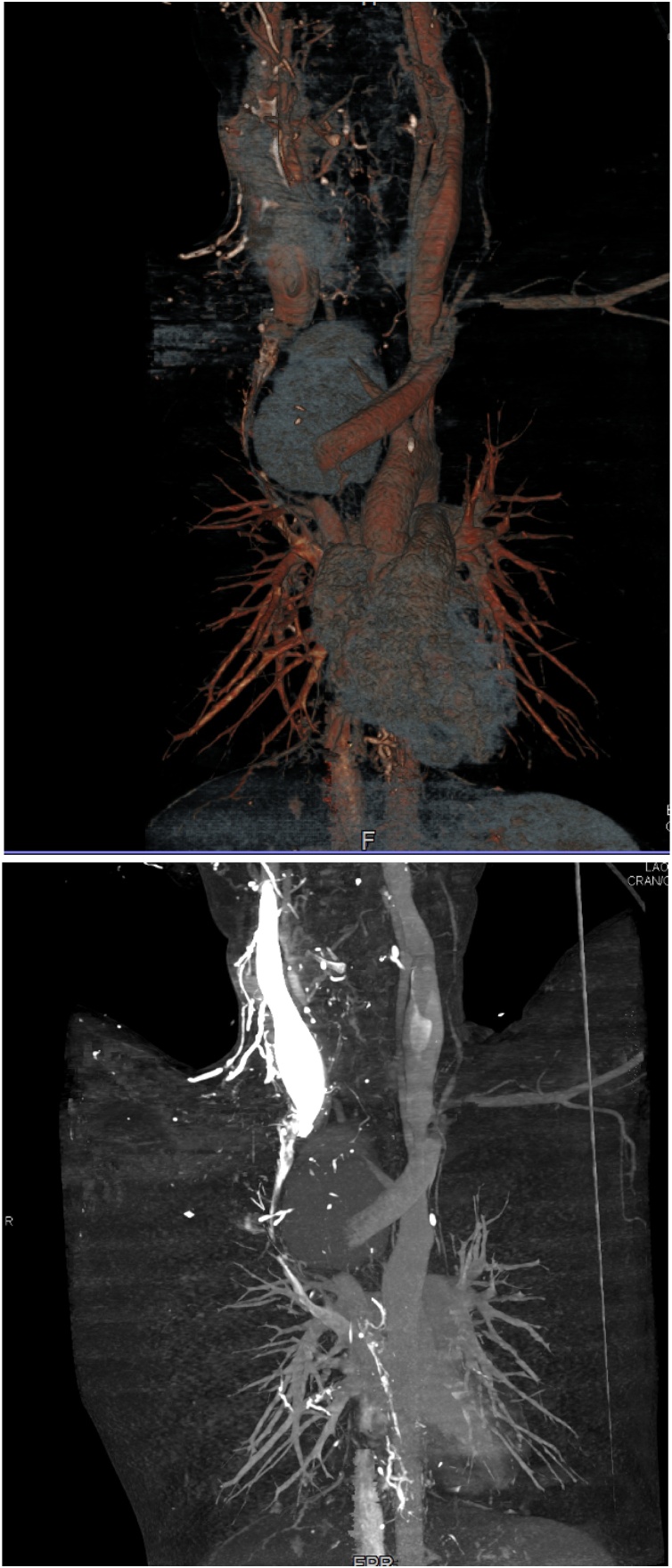
CT reconstruction images show the interruption of the innominate artery with the pseudoaneurysm. The right subclavian artery is not filling. The SVC is markedly compressed and thinned out.

A 24-year-old male patient was referred to our ER as a case of mediastinal tumor compressing the airways. On arrival, the patient was in severe respiratory distress; he had stridor, tachypnea, and marked desaturation (Pao2 less than 60%). The patient was not accompanied by any family member; thus, obtaining history was not possible at that point. The chest x-ray done at the referring hospital showed a huge right paratracheal mediastinal mass compressing the airways from the right side (Fig. 1). The patient was taken to the OR for intubation under fiberoptic bronchoscopy guidance. The fiberoptic bronchoscopic evaluation showed significant compression of the trachea from the right side with no endobronchial lesions. A small ETT (size 5) was successfully inserted. The patient was then taken to the radiology department for a chest CT with IV contrast. Chest CT showed a huge vascular mass (15 × 20 cm) consisting of a central pool of contrast with surrounding compressed tissue. The mass markedly compressed the trachea and the right mainstem bronchus with marked shifting of the mediastinum to the left side (Figs. 2 & 3). The SVC was markedly compressed, thinned out, and shifted by the pseudoaneurysm to the right side. The study showed the proximal 2 cm of the innominate artery, followed by the pseudoaneurysm. The right common carotid can be seen at the upper end of the pseudoaneurysm. There was an interruption of the right subclavian artery at its origin (Figs. Figs. 4 & 5). Collaterals were seen supplying the right upper limb.

The patient was taken immediately to the OR, and the mediastinum was explored through a midline sternotomy. A huge saccular aneurysm was identified arising from the lateral wall of the innominate artery with a fibrous capsule firmly adherent to the surrounding structures. The aneurysm was dissected from the surroundings, resected, and the innominate artery's lateral wall was repaired at the origin of the right common carotid artery. The distal end of the subclavian artery was arising from the far end of the pseudoaneurysm. We ligated the distal end of the right subclavian artery. Postoperatively, history was taken from the patient. He had a penetrating chest injury by a sheep horn in the right infraclavicular region 15 years prior to presentation. The patient's postoperative course was uneventful, and his respiratory symptoms resolved. The right upper limb was properly functioning, and CT angiography showed excellent filling of the right subclavian artery from the collateral circulation. Pseudoaneurysm of the innominate artery should be suspected as a rare complication in chest trauma.

## 3 Discussion

Pseudoaneurysm of the brachiocephalic artery is a very rare condition, with the majority occurring post chest trauma [1]. Other causes include infection, iatrogenic events following irradiation, or due to malignancy [4].

The clinical presentation may be early shortly after an incident or delayed; appearing years later. Some reported cases presented 20–30 years after the initial injury [1], [3]. The acute presentation of a thoracic great vessel injury is usually dramatic, with massive hemothorax and hemorrhagic shock [2]. The delayed presentations are usually nonspecific and may vary from mild respiratory distress, mimicking bronchial asthma, up to severe airway obstruction [5], [6]. Other delayed manifestations may include dysphagia, hemoptysis, hematemesis, pulse and neurological deficits, bruits, or cardiac failure [3]. This work has been reported in line with the SCARE 2018 criteria [9].

This case is particularly unique and unusual due to the significantly prolonged interval (15 years) between the traumatic event and the urgent clinical presentation; requiring life-saving surgical intervention.

Old trauma history should be taken seriously into account as it is the most common missed cause. Pseudoaneurysms are usually caused by penetrating chest injuries more frequently than blunt injuries. CT angiogram is the most accurate diagnostic tool that sets the pathway for a well-planned surgical intervention.

## Conflicts of interest

All Authors deny any conflict of interest.

## Funding

There were neither sponsors nor special funding for writing or publishing this case report.

## Ethical approval

Approval has been granted by the Clinical Research Committee.

## Consent

The related patient’s written and verbal consent was taken.

## Author contribution

Bader M. Tahlawi: Wrote the Manuscript. Collected the data for the case report.

Mohamed Regal: Operated the patient, Review the manuscript.

Amirah Hassan: Data collection.

## Registration of research studies

Not applicable.

It is a case report.

## Guarantor

Mohamed Ragal.

## Availability of data and materials

The datasets used during the current study are available from the corresponding author on reasonable request.

## Provenance and peer review

Not commissioned, externally peer-reviewed.
